# 

**Published:** 1894-01

**Authors:** 


### CONTENTS.

ORIGINAL COMMUNICATIONS—

The Diagnosis and Treatment of Movable Kidney. By J. B. S. Holmes, M. D., Atlanta, Ga. .......... 641
Enterectomy—End to End Anastomosis—Cholecyst-Enterostomy—With Murphy Button. By W. B. Rogers, M. D., Memphis, Tenn. .......... 648
Persistent Papillary Membrane Associated with Atrophy of Choroid and Optic Nerve. By S. Latimer Phillips, M. D., Savannah, Ga. .......... 652
The Diazo-Reaction in Typhoid Fever. By Julius Freidenwald, M. D., Baltimore... 655

SOCIETY REPORTS—
Orleans Parish Medical Society .......... 658

CORRESPONDENCE—
New York Letter .......... 666
An Interesting Case .......... 671

EDITORIAL—
The Defeat of the Bill .......... 673
The Doctor as a Business Man .......... 674
Dermatology .......... 675

SELECTIONS AND ABSTRACTS—
Skin Diseases and Syphilis .......... 677
General Medicine and Therapeutics .......... 682
Gynecology and Obstetrics .......... 687
Surgery .......... 696

MEDICAL ITEMS .......... 702
BOOK REVIEWS .......... 703
MAGAZINES RECEIVED .......... 704
ADVERTISERS' INDEX TO ADVERTISEMENTS .......... xii
CLASSIFIED INDEX TO ADVERTISEMENTS .......... xxxvii
ITEMS OF INTEREST .......... x, xi, xii
JOURNAL FOR 1894 .......... xxii
PREMIUM OFFERS .......... xxxvi

